# Criterion Validity of the Isometric Contraction Test for Diagnosis of Axis I DC/TMD Considering Specific Muscles

**DOI:** 10.1111/joor.70011

**Published:** 2025-06-25

**Authors:** Marcos Iglesias‐Peón, Juan Mesa‐Jiménez, César Fernández‐de‐las‐Peñas, Carmen Iglesias Peón, Nuria García Iglesias, María Bienvenido Villalba, Daiana Priscila Rodrigues‐de‐Souza, Francisco Alburquerque‐Sendín

**Affiliations:** ^1^ Doctoral Program in Biomedicine University of Córdoba Córdoba Spain; ^2^ Osteopatía y Fisioterapia Guadalajara Spain; ^3^ Department of Physical Therapy Universidad san Pablo CEU Madrid Spain; ^4^ Department of Physical Therapy, Occupational Therapy, Rehabilitation and Physical Medicine Universidad Rey Juan Carlos Madrid Spain; ^5^ Guadalajara University Hospital Guadalajara Spain; ^6^ Department of Nursing, Pharmacology and Physical Therapy Faculty of Medicine and Nursing, University of Córdoba Córdoba Spain; ^7^ Maimonides Biomedical Research Institute of Cordoba (IMIBIC) Córdoba Spain

**Keywords:** facial pain, muscle contraction, muscle disorders, oral medicine, temporomandibular disorders, validation of results

## Abstract

**Background:**

To determine the validity of the Isometric Contraction Test (ICTest) for the DC/TMD in axis I regarding specific muscles such as the masseter, temporalis and medial pterygoid muscles.

**Methods:**

Forty patients with a diagnosis of TMD axis I of muscle origin and forty healthy controls were evaluated with the ICTest. Sensitivity, specificity, positive predictive value (PPV), negative predictive value (NPV), positive likelihood ratio (LR+) and negative likelihood ratio (LR−) were determined concerning the DC/TMD on axis I of muscle type, individually assessing the masseter, temporalis and medial pterygoid muscles.

**Results:**

The ICTest achieved a sensitivity ranging from 41% to 88.9% in masseter and temporalis muscles with lower values for the medial pterygoid muscle and when referred myofascial pain subtype was analysed. Specificity remained stable in both situations, the subtype of myalgia and the muscles studied, ranging from 93.4% to 98.7%. The LR values indicated high variability depending on the muscle and type of pain, although LR+ and LR− were ≥ 7.08 and ≤ 0.76%, respectfully, in all cases.

**Conclusions:**

The ICTest is highly specific when studying the masseter, temporalis and pterygoid muscles, with the latest one showing the poorest results. Referred myofascial pain exhibits the lowest validity for all the muscles.

## Graphical abstract

This study investigates the criterion validity of the Isometric Contraction Test (ICTest) for the diagnosis of Axis I DC/TMD, with a focus on specific masticatory muscles. The test yielded higher diagnostic accuracy for the masseter and temporalis muscles compared to the medial pterygoid, particularly when assessing the referred myofascial pain subtype.

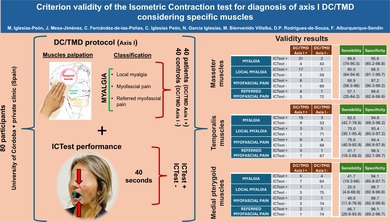

## 1 Background

Influenced by current neuroscience paradigms and modern diagnostic instruments [1], the Diagnostic Criteria for Temporomandibular Disorders (DC/TMD) [2] updated, in 2014, the existing clinical entities and incorporated new diagnoses. The DC/TMD on axis I classifies TMD based on the presence of muscle pain, joint pain or headache attributed to TMD, with muscle origin pain being the most prevalent, representing 45.3% of the diagnoses [3]. Muscle‐related TMD is classified into four subtypes: myalgia, tendinosis, myositis and muscle spasm [2]. Within myalgia, a new classification includes three subtypes: local myalgia, myofascial pain and referred myofascial pain [2], with the aim to provide a better precision in the diagnosis of muscle pain but also opening new challenges.

Even though the DC/TMD on axis I for muscle origin pain has demonstrated high validity [2], several aspects need further research. Good sensitivity and specificity data have been found for myalgia (sensitivity: 0.90, 95% CI 0.87–0.94; specificity: 0.99, 95% CI 0.97–1.00) and referred myofascial pain (sensitivity: 0.86, 95% CI 0.79–0.84; specificity: 0.98, 95% CI 0.97–0.99). On the contrary, the criterion validity for local myalgia and myofascial pain has not been described. Their content validity have been documented; however, no data are available for other masticatory muscles beyond masseter and temporalis, where palpation accessibility is more challenging [4]. The palpation of other masticatory muscles and structures such as the lateral pterygoid, medial pterygoid, temporal tendon, submandibular region and posterior mandibular region, that were excluded from the exploration due to their complex reach, did not provide any improvement in the validity or reliability of the DC/TMD [5, 6, 7].

These aspects justify questions regarding the methodology of the DC/TMD of muscle origin. The influence and experience of the examiner on manual palpation, the most efficient pressure time, the difficulty of exploring some muscles such as the posterior digastric or the medial pterygoid [7] and the limited functionality of the DC/TMD [8] are some of the issues that need to be investigated. Further limitations include the overlap of anatomical structures such as the deep part of the masseter muscle and the anterior portion of the joint capsule [9], or the correlation between the mechanical sensitivity to pain caused by the own palpation and the anatomical design of the masseter and temporalis muscles. This situation could evoke a hyperalgesic response in both healthy subjects [10] and patients [11].

New methodologies to evaluate TMD of muscle origin have appeared in the last years. The Isometric Contraction Test of the Masticatory Muscles (ICTest) [12] has demonstrated good results in validity and reliability, and also a high functionality and small assessor dependence [12, 13]. This test allows to explore other muscles in addition to the masseter and temporalis, involved in mouth closure, such as the medial pterygoid muscles, that are also relevant in the DC/TMD [14]. However, the ICTest has not been validated by muscle units, a situation which could improve the prognosis and therapeutic approach. Therefore, the present study aims to determine the validity of the ICTest by masticatory muscle units, that is, temporalis, masseters and medial pterygoids muscles and compared to the DC/TMD.

## 2 Methods

### 2.1 Design

A criterion validity study for the ICTest by masticatory muscle units with a non‐probabilistic recruitment of consecutive cases and controls was performed. Each participant signed an informed consent to be included in the study. The University of Córdoba's ethics committee (code 5372 2022) approved the study protocol.

### 2.2 Participants

Eighty subjects were selected from a total of one hundred twenty‐two individuals at the Health Campus of the University of Córdoba and a private clinic in Guadalajara (Spain). Participant identification was conducted in three ways: patients from the private clinic reporting TMD pain; individuals with a previous history of TMD who contacted at any of the study centres; and subjects who took part in requests or announcements on social networks.

Inclusion criteria for the case group were as follows: age between 20 and 65 years, any gender and pain or headache related to the masticatory muscles in the last thirty days, complying with the DC/TMD on axis I of muscle origin. On the other hand, the control group included subjects who have never had pain or have not experienced it in the last thirty days and therefore did not follow the DC/TMD on axis I of muscle origin. The exclusion criteria were: being edentulous, having acute dental pathologies that do not allow a maximum contraction of the masticatory muscles, experiencing issues in understanding the instructions, suffering from joint blockage in the temporomandibular joint (TMJ) or having undergone a TMJ intervention in the last thirty days. To avoid significant asymmetries between groups, subjects in the control group were matched with cases based on sex, age (±5 years) and Body Mass Index (BMI, ±3 kg/m^2^) [15]. Fourteen patients had previously participated in earlier research of the ICTest [12]. To avoid any potential sources of bias, a minimum interval of 6 months was maintained between the two evaluations.

### 2.3 Procedures

**FIGURE 1:**
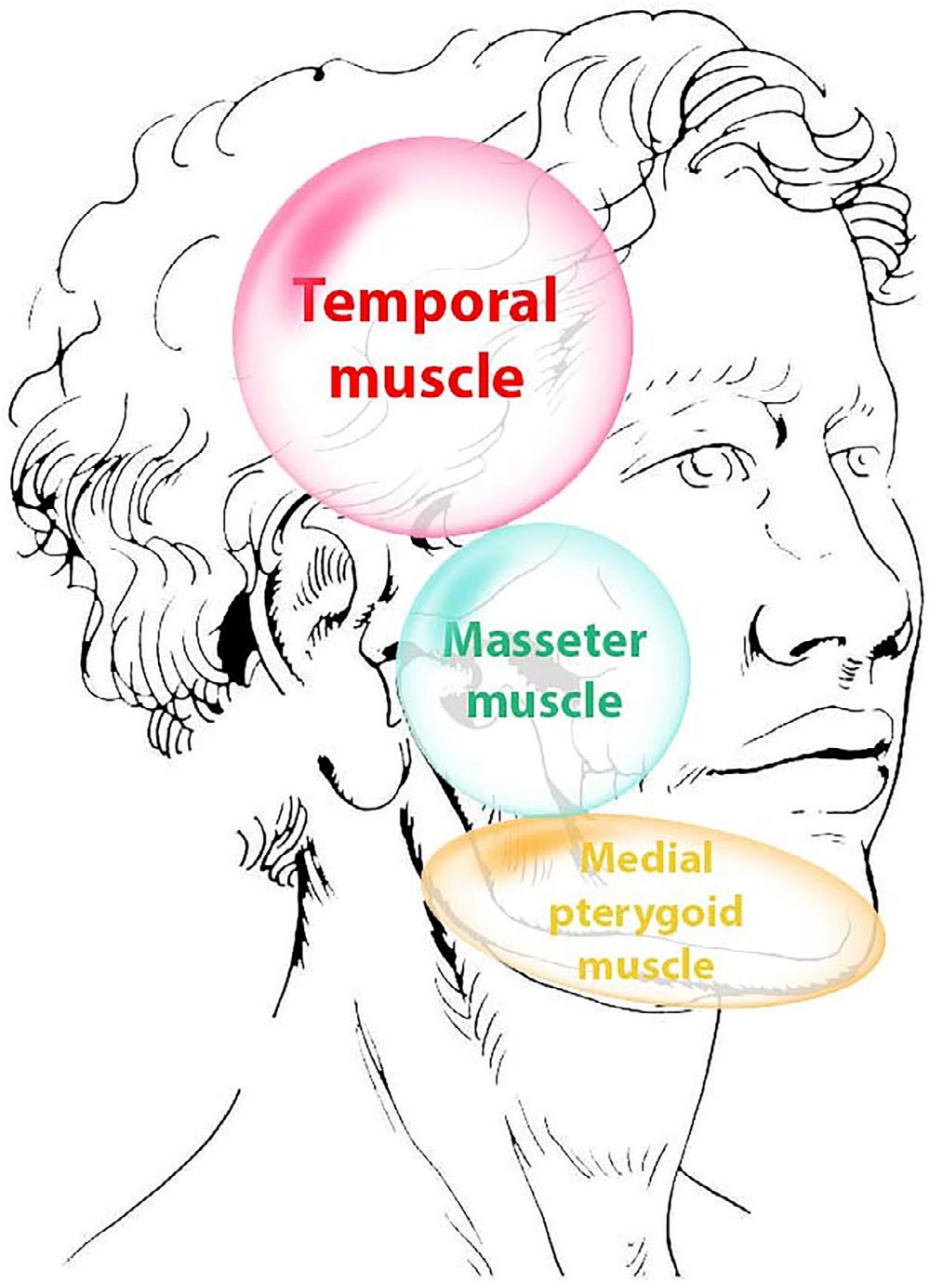
Myofascial pain map for temporalis, masseter and medial pterygoid muscles.

Participants underwent the DC/TMD according to the validated Spanish version [16, 17]. A physiotherapist with more than 5 years of experience in managing orofacial and temporomandibular pain performed the DC/TMD Axis I evaluation. Accordingly, the participants completed the Symptom Questionnaire (Symptom Questionnaire DC/TMD SQ) which divided participants into cases (DC/TMD Axis I +) if they answered affirmatively to items 3 and 4, or controls (DC/TMD Axis I−) if they did not. Once completed this first questionnaire, the next step of the diagnostic algorithm of the DC/TMD began with the Examination Form [16]. In this form, the examiner verbally confirmed painful areas as the patient corroborated that they were experiencing their symptoms (like the pain felt in the last thirty days). Afterward, a bilateral palpation was performed at the points described in the Examination Form, examining one side at a time [16]. If manual palpation triggered familiar pain (reproduced the symptoms) on the temporalis or masseter muscles, then that hurt was diagnosed as myalgia. The anatomical characteristics then differentiated the subtypes of pain in local myalgia, myofascial pain or referred myofascial pain [12, 13, 16]. Additionally, manual examination of the medial pterygoid muscle was performed at the lower angle of the jaw on its inner face [18, 19, 20, 21, 22]. The muscle pain maps of each muscle [19, 22] were considered in their assessment. To distinguish the pain originated among the muscles, the referred pain located in the temporal fossa, even if pain appears in other cephalic areas, was considered referred pain from the temporalis muscle; referred pain from the masseter muscle was located in the teeth, mainly upper and lower molars, and pain in the cheek, although pain in the TMJ or the supraorbital arch may also occur; referred pain from the medial pterygoid muscle was located in the ascending ramus of the mandible, described as deep or non‐superficial pain, although pain in the TMJ may also occur (Figure 1) [19].

**FIGURE 2:**
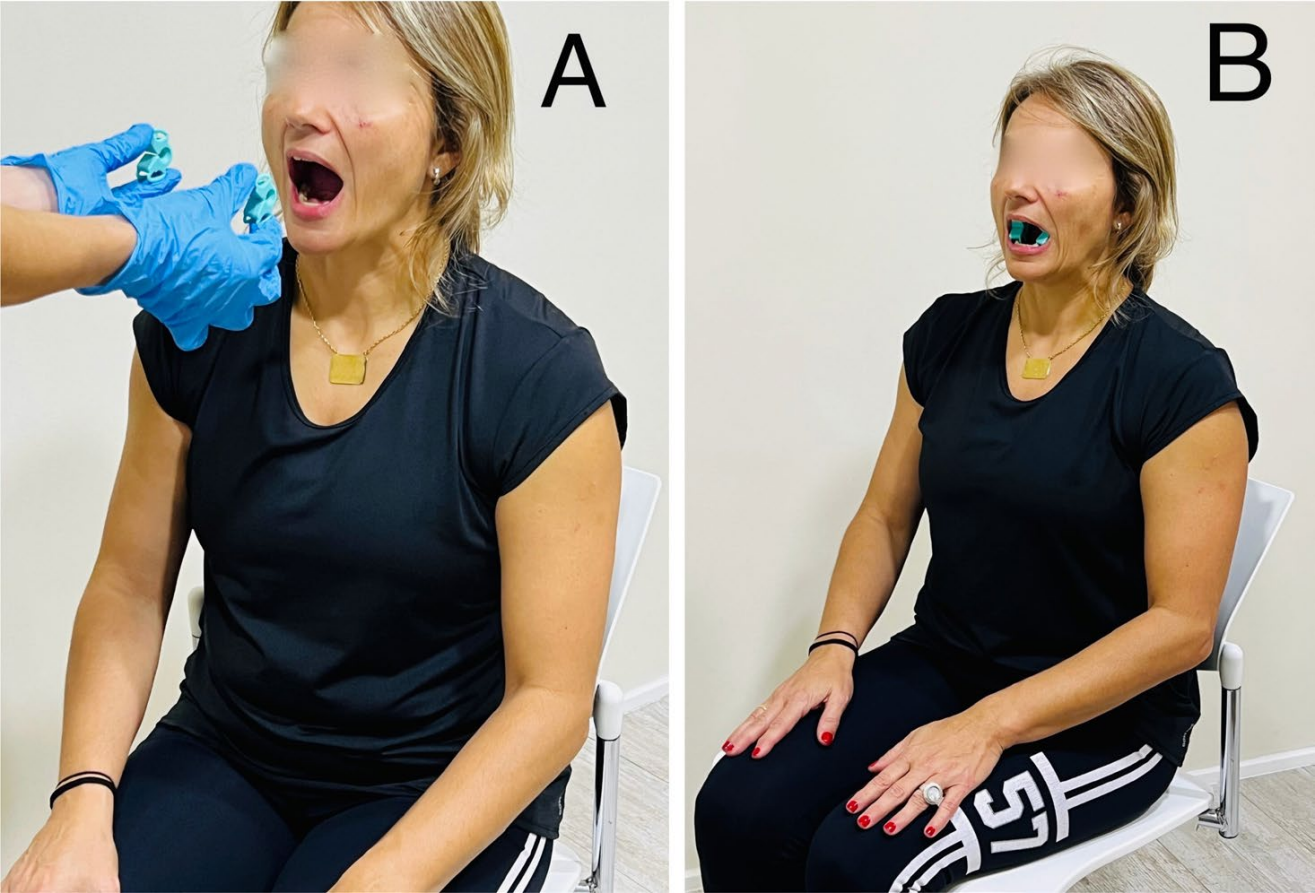
Isometric contraction test of the occlusive masticatory muscles. (A) First, the subject opens the mouth, and the assessor inserts the teethers in the subject's mouth; (B) Second, the subject clenches the teeth for forty seconds.

Five minutes later, another physiotherapist, trained for two hours on the ICTest, unaware of the DC/TMD results [12], applied the standardised ICTest protocol as described in previous studies [12, 13]. Participants were instructed to indicate any painful point or area during the exploration and to report any limiting pain that could interrupt the test. Two bite blocks teethers model (Barder laboratory, reference 11/022), with dimensions 3.3 × 2.8 × 1.8 cm), were placed bilaterally and symmetrically between the lower molars and premolars. The correct positioning of both bite blocks was monitored throughout the entire development of the test. Then, the individuals performed a maximum occlusive contraction for forty seconds, supervised by the examiner, with the aim of activating all muscle units under fatigue [23, 24, 25] (Figure 2). A final collection of pain data was fulfilled, that is, if and where the patient experienced pain, and if it could be classified as a familiar pain. The test was considered positive if the latest requirement was met. Otherwise, the test was considered negative. In the presence of a positive test, the pain was considered myalgia and thus subdivided according to the anatomical distribution and published maps [14, 19, 22], analogous to the DC/TMD.

### 2.4 Sample Size

To calculate the sample size, it should be taken into consideration that when both the muscle and the type of muscle pain are studied, possible imbalances of sensitivity and specificity may appear, as previously reported [12]. Thus, according to a comparison of two proportions calculated with a chi‐squared test with continuity correction [26], to detect a sensitivity of at least 0.5 and a specificity of 0.9, with a two‐tailed test and a proportion between cases and controls of 1:1, 72 subjects are required. Finally, considering a 10% loss of data in each group, the sample was increased to 40 subjects per group, and therefore, 80 subjects in total were calculated as an appropriate sample.

### 2.5 Statistical Analysis

The quantitative data were described as mean, standard deviation, and 95% CI, while frequencies and percentages were used for categorical data. The demographic data followed a normal distribution, according to the Kolmogorov–Smirnov test, in both groups. Unpaired two‐tailed student's *t*‐tests were used to identify differences in the sociodemographic data of both groups.

To test the criterion validity of the ICTest compared with the DC/TMD of axis I as the gold standard for the masseter, temporalis and internal pterygoid muscles, sensitivity, specificity, positive (PPV) and negative predictive values (NPV), in all cases expressed as percentage, and positive (LR+) and negative likelihood ratios (LR−), were obtained [27, 28]. To interpret the performance of the ICTest, a sensitivity or specificity > 0.8 [28], as well as LR+ > 10 and LR− < 0.1 [29], were considered good. The Youden index, which integrates sensitivity and specificity [30], was acceptable when ≥ 0.5 [31].

A 95% CI was calculated for each statistical test. Significant *p* values were considered when *p* < 0.05. Data were analysed with the IBM‐SPSS version 28.0.

## 3 Results

**FIGURE 3:**
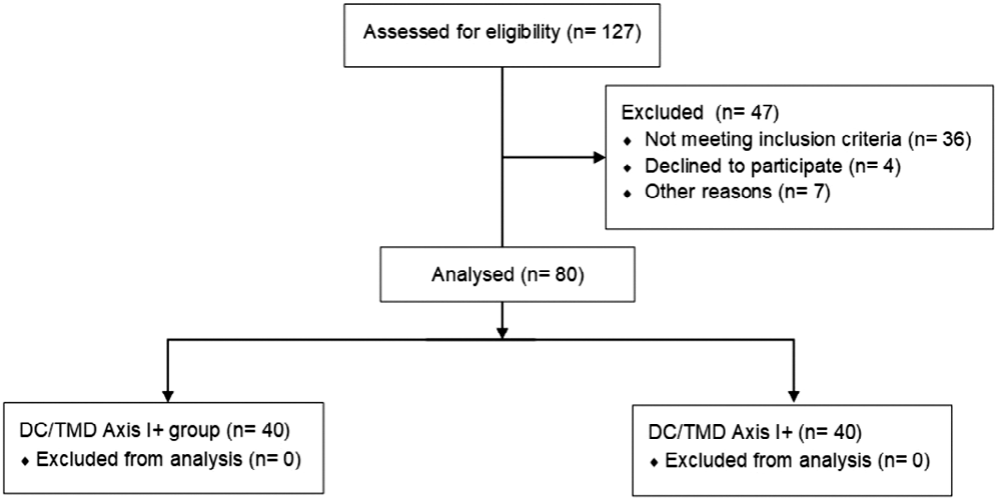
Flow chart of the study.

Eighty subjects were divided into two groups. Forty (40% men, mean age: 45.8 ± 13.9 years, BMI: 24.5 ± 3.2 kg/m^2^) with a symptom history of 7.4 ± 5.1 years were included in the DC/TMD Axis I+ group. Another 40 (40% male, age: 47.6 ± 11.8 years, BMI: 24.1 ± 2.6 kg/m^2^) participants formed the DC/TMD Axis I‐. No individual had to abandon the study protocol at any time or reported unbearable pain group (Figure 3).

The ICTest results varied depending on the muscle studied and the type/subtype of TMD evaluated. Thus, masseter muscles presented the highest validity percentages (≥ 80%) for myalgia and its subtypes, except for the sensitivity value observed on referred myofascial pain subtype. In fact, the Youden index for the masseter muscles was acceptable in all cases (≥ 0.6), and the LR+ consistently showed values above 19, while the LR− was not lower than 0.10 in any case (Table 1).

**TABLE 1:** Validity results for the ICTest with respect to the masseter muscles according to the DC/TMD Axis I (myalgia and myalgia subtypes).

		DC/TMD Axis I+	DC/TMD Axis I−	Total	Sensibility	Specificity	PPV	NPV	Youden index	LR+	LR−
OVERALL MYALGIA	ICTest+	31	2	33	88.6 (74–95.5)	95.6 (85.2–98.8)	93.9 (80.4–98.3)	91.5 (80.1–96.6)	0.8	19.93 (5.11–77.65)	0.12 (0.05–0.30)
	ICTest−	4	43	47							
	TOTAL	35	45	80							
LOCAL MYALGIA	ICTest+	17	1	18	80.0 (64–94.8)	98.3 (91.1–99.7)	94.4 (74.2–99)	95.2 (86.7–98.3)	0.8	51.00 (7.2–349.2)	0.15 (0.05–0.43)
	ICTest−	3	59	62							
	TOTAL	20	60	80							
MYOFASCIAL PAIN	ICTest+	8	2	10	88.9 (56.5–98)	97.2 (90.3–99.2)	80.0 (49–94.3)	98.6 (92.3–99.7)	0.9	31.56 (7.89–126.14)	0.11 (0.02–0.73)
	ICTest−	1	69	70							
	TOTAL	9	71	80							
REFERRED MYOFASCIAL PAIN	ICTest+	4	1	5	57.1 (25–84.2)	98.6 (92.6–99.8)	80.0 (37.6–96.4)	96.00 (88.9–98.6)	0.6	41.71 (5.37–323.88)	0.43 (0.18–1.03)
	ICTest−	3	72	75							
	TOTAL	7	73	80							

*Note:* Expressed as value (95% confidence interval).

Abbreviations: NPV: negative predictive value; PPV: positive predictive value; sensitivity, specificity, PPV, NPV expressed as percentage (95% confidence interval). LR +: Positive likelihood ratio. LR−: Negative likelihood ratio.

Sensitivity in the temporalis muscles did not achieve 80% in any type of muscle pain. The lowest values corresponded to referred myofascial pain. Specificity values reached high values (≥ 93.4%) with a peak percentage seen in referred myofascial pain. For the temporalis muscle, referred myofascial pain was the only one that did not achieve an acceptable Youden index (Table 2). The likelihood ratios followed a similar pattern to what was observed in the masseter muscles but with less pronounced values. Once again, LR+ fell into the category of ‘Good’ in all cases (> 10), whereas no type of muscle pain showed a LR− < 0.20 (Table 2).

**TABLE 2:** Validity results for the ICTest with respect to the temporalis muscles according to the DC/TMD Axis I (myalgia and myalgia subtypes).

		DC/TMD Axis I+	DC/TMD Axis I−	Total	Sensibility	Specificity	PPV	NPV	Youden index	LR+	LR−
OVERALL MYALGIA	ICTest+	15	3	18	62.5 (42.7–78.8)	94.6 (88.5–98.2)	83.6 (60.8–94.2)	85.5 (74.7–92.2)	0,6	11.67 (3.72–36.61)	0.40 (0.23–0.67)
	ICTest−	9	53	62							
	TOTAL	24	56	80							
LOCAL MYALGIA	ICTest+	3	5	8	75.0 (30.1–95.4)	93.4 (85.5–97.2)	37,5 (13.7–69.4)	98.6 (92.5–99.8)	0,7	11.40 (4.12–31.58)	0.27 (0.05–1.48)
	ICTest−	1	71	72							
	TOTAL	4	76	80							
MYOFASCIAL PAIN	ICTest+	6	4	10	75.0 (40.9–92.9)	94.4 (86.6–97.8)	60.0 (31.3–83.2)	97.1 (90.2–99.2)	0,7	13.50 (4.81–37.93)	0.26 (0.08–0.89)
	ICTest−	2	68	70							
	TOTAL	8	72	80							
REFERRED MYOFASCIAL PAIN	ICTest+	5	1	6	41.7 (19.3–68.0)	98.5 (92.1–99.7)	83.3 (43.6–97)	90.5 (81.7–95.3)	0.4	28.33 (3.62–221.75)	0.59 (0.36–0.96)
	ICTest−	7	67	74							
	TOTAL	12	68	80							

*Note:* Expressed as value (95% confidence interval).

Abbreviations: NPV: negative predictive value; PPV: positive predictive value; sensitivity, specificity, PPV, NPV expressed as percentage (95% confidence interval). LR +: Positive likelihood ratio. LR−: Negative likelihood ratio.

Finally, the medial pterygoid muscles were affected in only twelve out of eighty subjects and showed the poorest sensitivity data for myalgia and its subtypes (25%–66.7%). On the contrary, their specificity consistently surpassed 94%. The PPV did not reach 80% in any case, while the NPV remained above 90%. Consequently, only the Youden index for referred myofascial pain subtype resulted acceptable (Table 3). Similar to sensitivity and PPV, LR− showed its highest values (lower diagnostic capacity), as well as the LR+ for myalgia, which was the lowest in the study, not reaching the threshold of ‘good’ (Table 3).

**TABLE 3:** Validity results for the ICTest with respect to the medial pterygoid muscles according to the DC/TMD Axis I (myalgia and myalgia subtypes).

		DC/TMD Axis I+	DC/TMD Axis I−	Total	Sensibility	Specificity	PPV	NPV	Youden index	LR+	LR−
OVERALL MYALGIA	ICTest+	5	4	9	41.7 (19.3–68)	94.1 (85.8–97.7)	55.6 (26.7–81.1)	90.1 (81–95.1)	0.4	7.08 (2.21–22.66)	0.62 (0.37–1.03)
	ICTest−	7	64	71							
	TOTAL	12	68	80							
LOCAL MYALGIA	ICTest+	1	1	2	25.0 (4.6–69.9)	98.7 (92.9–99.8)	50.0 (9.5–90.5)	96.2 (89.3–98.7)	0.2	19.00 (1.44–252.5)	0.76 (0.42–1.37)
	ICTest−	3	75	78							
	TOTAL	4	76	80							
MYOFASCIAL PAIN	ICTest+	2	1	3	40.0 (11.8–76.9)	98.7 (92.8–99.8)	66.7 (20.8–93.9)	96.1 (89.2–98.7)	0.4	30.00 (3.25–277.1)	0.61 (0.29–1.26)
	ICTest−	3	74	77							
	TOTAL	5	75	80							
REFERRED MYOFASCIAL PAIN	ICTest+	2	3	5	66.7 (20.8–93.9)	96.1 (89.2–98.7)	40.0 (11.8–76.9)	98.7 (92.8–99.8)	0.6	17.11 (4.36–67.19)	0.35 (0.07–1.74)
	ICTest−	1	74	75							
	TOTAL	3	77	80							

*Note:* Expressed as value (95% confidence interval).

Abbreviations: NPV: negative predictive value; PPV: positive predictive value; sensitivity, specificity, PPV, NPV expressed as percentage (95% confidence interval). LR+: Positive likelihood ratio. LR−: Negative likelihood ratio.

## 4 Discussion

This study suggests that the ICTest can be a valid tool for the diagnosis of muscle pain and the assessment of masticatory muscle units separately, particularly in terms of specificity and LR+ if compared to the DC/TMD Axis I of muscle origin. Specifically, the test showed high sensitivity and specificity values for the masseter muscles but limited ability to detect cases of referred myofascial pain. For the temporalis and medial pterygoid muscles, the ICTest exhibited a high probability of positive diagnosis for any type of muscle pain. Its high specificity and LR+, except for medial pterygoid muscle myalgia, means that a positive result can confirm the presence of TMD, while the absence of positivity does not exclude it. The fact that none of the 80 participants dropped out in this study supports its applicability in a clinical setting.

The ICTest shows a higher specificity than sensitivity for all muscles and subtypes of pain. This pattern aligns with several features associated with the evaluation of muscle pain, also present in the DC/TMD for myalgia and referred myofascial pain [2], and even in the assessment of myofascial pain in the upper quarter musculature [32]. In contrast, higher sensitivity than specificity is identified in the analysis of cervical hypomobility and other areas [33]. The elevated specificity and LR+ of the ICTest, especially for the masseter and temporalis muscles, indicate that a positive result would confirm the presence of TMD. On the other hand, its lower sensitivity would not allow one to dismiss its presence if the result were negative. It is crucial that in a diagnostic process, different examinations are applied [34, 35], beginning with those with higher sensitivity and concluding with those with higher specificity, such as the ICTest. This enables one to surpass the diagnostic threshold and would facilitate the beginning of any treatment.

The utility of the ICTest relies on avoiding dependence on examiner palpation by incorporating a functional muscle assessment, such as occlusal force, to diagnose muscle TMD [12]. There is no justified consent for choosing a palpation that lasts two versus five seconds or the duration of the period between palpation procedures [7]. This matter could lead to detecting false positives (apparent pathological patterns in healthy individuals) [36]. Additionally, errors could also derive from the fact that certain parts of the masticatory muscles, such as the posterior angle of the mandible for the medial pterygoid, are not easily accessible to deep palpation, which is used as a supplementary evaluation technique [7, 9, 37]. Palpation on the lateral pole of the TMJ has also been questioned, since anatomically, the deeper part of its structure coincides with the anterior part of the TMJ capsule [9]. Likewise, the anatomical design of the temporalis and masseter muscles may lower the pain threshold if pressure is applied on them, evoking pain in individuals with [10] and without TMD [11]. Considering the medial pterygoid muscle, even though it is difficult to palpate [18, 34, 38], it plays a crucial role in mandibular stabilisation in the vertical and horizontal planes [39, 40], and is part of various TMJ pathologies and headaches [6, 14, 16, 36, 39, 41]. Results obtained in this study indicate that the presence of pain in the medial pterygoid muscle during occlusion is highly indicative of TMD of muscular origin, especially for its subtypes. However, these results should be considered with caution due to the low prevalence (15%) in the patient sample with DC/TMD Axis I + for this muscle [2, 3, 32, 33].

The ICTest does not require previous experience and training to be clinically applied successfully [13], which is considered a positive aspect for a clinical test. A rigorous calibration of the assessors could be seen as a relevant requirement to obtain high reliability of each subgroup of DC/TMD [7]. In fact, the amount of training necessary to adequately use DC/TMD has been reported as a cause of its limited clinical implementation [42]. However, this is controversial, since longitudinal studies have revealed no substantial changes of reliability when disagreements between assessors, related to their experience, occur [43].

Multiple reasons, such as the complex understanding of referred myofascial pain [44], could justify why it yields the poorest results in terms of validity [12] and reliability [13] when compared to the other types of pain. This problem has also emerged in the validation of the DC/TMD in axis I in children, probably due to the complexity of understanding this type of pain and its description [44]. Even it has also been suggested that this pain form could be significantly related to a component of the DC/TMD on axis II [45], supported because patients with local myalgia subtype of TMD obtain lower values of depression, anxiety, somatic symptoms, catastrophising, perceived stress and insomnia compared to patients with myofascial pain and referred myofascial pain subtypes [46]. In fact, these two types of pain have been linked to central sensitisation [47], chronic TMD [48, 49], or fibromyalgia syndrome, related to a central impairment of maladaptive learning nature, neuronal plasticity secondary to a peripheral dysfunction, and even to genetic polymorphisms associated with hormones [50]. These motives increase the difficulty of obtaining valid results in referred myofascial pain diagnosis and support the inclusion of psychosocial criteria to diagnose this subtype of muscle distress.

The following limitations should be acknowledged in the current study. The ICTest has not been evaluated on individuals with missing teeth or dental pain that could affect mandibular occlusion. Altered pain states, such as fibromyalgia [46], central sensitisation [46, 49, 51], neck pain [35, 49, 52] and other biological aspects [50, 53], which could impact the ICTest results, especially for referred myofascial pain [33, 44, 47, 48, 49, 50, 51], were not considered. Additionally, the unilateral or bilateral nature of the conditions has not been addressed, and patients were not grouped based on the number of painful muscles. Further studies are needed to address these factors and to revise current diagnostic criteria according to new evidence [54]. It should also be noted that the difficulty of palpating the medial pterygoid muscle may have affected the accurate assessment prior to the ICTest.

## 5 Conclusion

The ICTest is highly specific according to the DC/TMD on axis I of muscle origin, for the masseter, temporalis and medial pterygoid muscles, with the latter showing the poorest results. Among the subtypes of myalgia, referred myofascial pain exhibits lower validity in the evaluated muscles. The ICTest is a useful tool, mainly to confirm the presence of muscle pain, added to the DC/TMD on axis I, due to its functional approach and the lack of need for prior training in palpation techniques.

## Conflicts of Interest

The authors declare no conflicts of interest.

## Data Availability

The data that support the findings of this study are available from the corresponding author upon reasonable request.
